# Invariant Distribution of Promoter Activities in *Escherichia coli*

**DOI:** 10.1371/journal.pcbi.1000545

**Published:** 2009-10-23

**Authors:** Alon Zaslaver, Shai Kaplan, Anat Bren, Adrian Jinich, Avi Mayo, Erez Dekel, Uri Alon, Shalev Itzkovitz

**Affiliations:** 1 Division of Biology, California Institute of Technology, Pasadena, California, United States of America; 2 Department of Molecular Cell Biology, Weizmann Institute of Science, Rehovot, Israel; 3 Department of Computer Science and Applied Mathematics, Weizmann Institute of Science, Rehovot, Israel; 4 Department of Biological Chemistry, Weizmann Institute of Science, Rehovot, Israel; University of Illinois at Urbana-Champaign, United States of America

## Abstract

Cells need to allocate their limited resources to express a wide range of genes. To understand how *Escherichia coli* partitions its transcriptional resources between its different promoters, we employ a robotic assay using a comprehensive reporter strain library for *E. coli* to measure promoter activity on a genomic scale at high-temporal resolution and accuracy. This allows continuous tracking of promoter activity as cells change their growth rate from exponential to stationary phase in different media. We find a heavy-tailed distribution of promoter activities, with promoter activities spanning several orders of magnitude. While the shape of the distribution is almost completely independent of the growth conditions, the identity of the promoters expressed at different levels does depend on them. Translation machinery genes, however, keep the same relative expression levels in the distribution across conditions, and their fractional promoter activity tracks growth rate tightly. We present a simple optimization model for resource allocation which suggests that the observed invariant distributions might maximize growth rate. These invariant features of the distribution of promoter activities may suggest design constraints that shape the allocation of transcriptional resources.

## Author Summary

Cells respond to a changing environment by regulating the activity of genes. Here, we sought to understand how *E. coli* cells distribute their limited transcriptional resources among their target genes, and how this allocation varies with growth rate and growth conditions. To achieve this, we assayed the expression of a comprehensive library of transcriptional reporter strains under different conditions. High-temporal resolution measurements of promoter activities were obtained for different growth rates spanning recovery from stationary phase into exponential phase and eventually deep stationary phase again. We find that the genome-wide promoter activity follows a power-law distribution, which depends solely on growth rate and is independent of the specific growth conditions. Moreover, we find that the power-law distribution can be decomposed into two log-normal distributions: metabolic promoters that make up the low end of the distribution, and ribosomal promoters that make up the high end of the distribution. While distributions remained constant for a given growth rate, the ranked expression of metabolic promoters differed according to the specific condition. Thus, the invariant distribution may suggest optimal resource allocation under constrained resources. A mathematical theory is presented to explain these results.

## Introduction

Bacteria face an interesting optimization problem: How to allocate limited transcriptional resources among thousands of different promoters. Beginning with the pioneering work of the Copenhagen school, several studies have measured the composition of the bacterial cell at different growth rates. Precise measurements were made of RNA, DNA, cell mass and size, as well as ribosome content [1]–[4]. These studies were performed in a handful of conditions at balanced growth (exponential phase), using methods such as sucrose gradient centrifugation [1] and RNA pulse labeling and hybridization [2]. It was found that growth rate is a key parameter determining cellular composition [1], [5]–[9]. Total DNA, RNA and cell size were found to increase with growth rate, while protein elongation rate and total protein concentration remain fairly constant. One of the important findings of these studies was that the ribosome fraction increases linearly with growth rate [3], [4], [10]–[14]. A recent study also demonstrated that partition of RNA polymerases dependes on growth rate as well [15]. To complement this work on general cell composition, one needs to measure the activity of individual promoters on a genome wide scale under diverse conditions and at different growth rates and stages of growth.

Here we study the transcriptional resource allocation in *E. coli* on a genomic scale. We used a robotic assay based on a recently described approach [16] to measure the promoter activity at high accuracy and temporal resolution in a variety of growth conditions. This approach allows tracking the promoter activity as a function of time as cells grow from exponential to stationary phase in diverse conditions.

We find that the distribution of promoter activities at a given growth rate is invariant to growth conditions. This distribution shows a heavy-tail, with promoter activities that span nearly four orders of magnitude. The distribution shape depends somewhat on growth rate: The higher the growth rate the more skewed the distribution. The distribution can be decomposed into at least two distinct classes of promoters showing different behavior between conditions: ribosomal promoters and metabolic promoters. The class of ribosomal promoters is invariably highly expressed in a correlated manner between conditions, while the promoters of metabolic proteins are expressed at low-intermediate levels and vary between different growth conditions. Fractional ribosomal promoter activity closely follows growth rate in the non-balanced growth conditions studied. We also study a simple optimization model for resource allocation, which suggests that the observed invariant distribution can maximize the growth rate.

## Results

### Dynamics of promoter activity on a genomic scale in *E. coli* under various growth conditions

We sought to measure the activity of *E. coli* promoters as a function of time in different conditions and phases of growth. To measure promoter activity we used a comprehensive library of 1,920 reporter strains, each of which contains a low-copy plasmid with a rapidly folding GFP variant fused to a copy of one of the cells' promoters. The promoter region on the plasmid includes the entire intergenic region. These cells turn green in proportion to the rate of transcription from the promoter. Moreover, the GFP is highly-stable and accumulates over time; Thus, promoter activities can be easily extracted by following the derivative of the fluorescent signal over time. Previous work indicated that this library can serve as an accurate tool for measuring promoter activities [16]–[18].

To obtain high-throughput measurements of the entire library under different growth conditions, we developed a new method using robotics. We used a robotic liquid handling system to inoculate the cells in 384-well plates, grow them in an automated incubator, and periodically transfer them to a multi-well fluorimeter/photometer. Cell density and fluorescence were measured at a 16 min resolution over 14 h of growth. In the resulting dataset, each promoter was assayed at 52 time points over the growth curve, which spanned exponential phase and entrance into stationary phase. Reproducibility of fluorescence at a given growth rate was high (coefficient of variance ∼20%, Fig S1).

**Figure 1:**
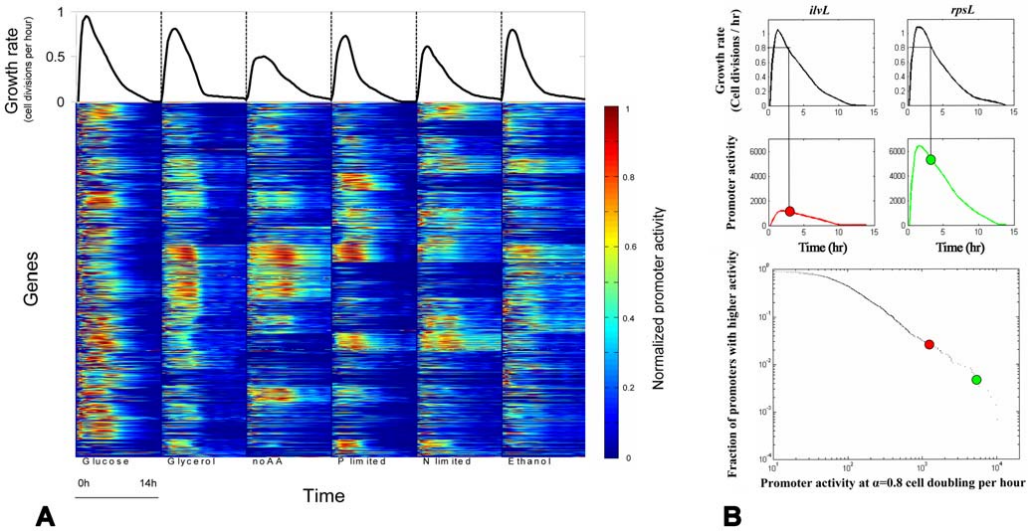
Genome-scale promoter activity assay at different growth rates. **(A)** Shown are the promoter activities of 1,920 promoters in *E. coli* (bottom) and their average growth rate (top) under six different growth conditions measured along 14 hours. Red represents high activity, blue represents low activity. Each expression pattern is normalized between zero and one where zero is the lowest expression level over all conditions and time-points and one is the highest. **(B)** Distribution of promoter activities can be extracted for each growth rate. The figure highlights two genes, *ilvL* and *rpsL*. Top-left curve is the growth rate of *ilvL* and top-right is the growth rate of *rpsL*. Bottom curves show the promoter activities of the two genes – *ilvL* (red) and *rpsL* (green). The promoter activity at the point where growth rate was 0.8 divisions per hour is indicated by red and green circles for *ilvL* and *rpsL* respectively. These values are shown on a rank-frequency plot of all promoter activities at the same growth rate, where the X-axis shows the promoter activity levels at a given growth rate and the Y-axis shows the fraction of promoters with equal or higher promoter activity levels at that growth rate. All plots are for the Glucose defined medium at 30°C.

The experiment was performed under several growth conditions (Table 1), that had different availability of carbon, nitrogen and other nutrients. These conditions resulted in different growth rates and final OD levels (Table 1). Note that these growth conditions imposed the cells to undergo continuous transient growth rates as opposed to steady-state balanced exponential growth (Fig 1A). In each condition, we found that different sets of promoters were expressed with differing intensities (Fig 1A). Each condition yielded data on the promoter activities of the cells at different stages of growth, from early exponential to deep stationary phases. We find that the sum of all promoter activities increases with growth rate but that at any given growth rate it is quite constant between conditions (Fig S2). We extracted the promoter activities corresponding to the different growth rates and plotted their distribution in a rank-frequency manner for further analysis (Fig 1B).

**Table 1:** Maximal OD and growth rates in various conditions.

Conditions	Maximal OD	Maximal growth rate (cell divisions per hour)*
Glucose	0.343 (2)	0.92(1)
Glycerol	0.199(1)	0.76(1)
No amino-acids	0.145(1)	0.54(1)
Phosphate limitation	0.139(1)	0.76(1)
Nitrogen limitation	0.129(1)	0.65(1)
Ethanol 4%	0.137(1)	0.76(1)

Numbers in parentheses are standard errors in last digit.

*: Maximal growth rate is the maximal growth rate which was reached by 90% of the strains.

### Invariant, heavy-tailed and scale rich distribution of promoter activities

**Figure 2:**
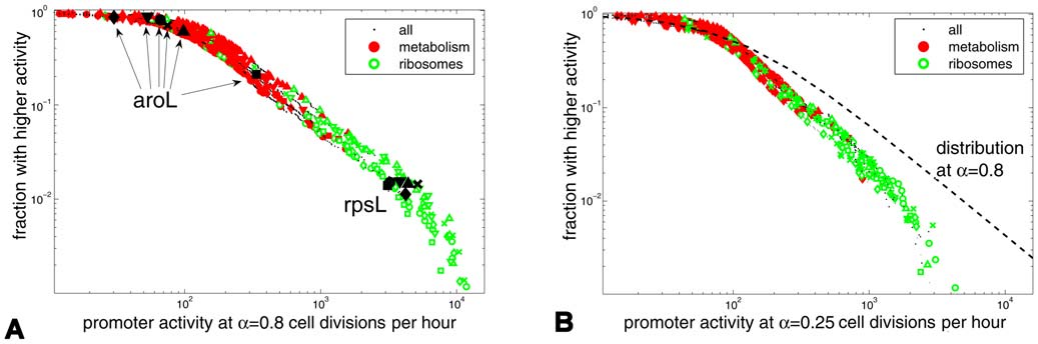
Invariant scale-rich distribution of promoter activities. **(A,B)** Rank-frequency plots of promoter activities for the six growth conditions of Fig 1. Horizontal axis shows promoter activity levels at a given growth rate; Vertical axis shows the fraction of promoters with an equal or higher promoter activity level. Black points – all genes; Empty green – ribosomal promoters; Solid red – metabolic proteins. X – glucose medium, circles – ethanol, diamonds – glycerol, squares – no amino-acids, V – Phosphate limitation, triangles – Nitrogen limitation. **(A)** Data at *α* = 0.8 cell divisions per hour. Black filled shapes: examples of an amino-acid biosynthesis promoter, *aroL*, the promoter activity levels of which vary widely between conditions and of a ribosomal promoter *rpsL*, the promoter activity levels of which are quite constant between conditions. **(B)** Data at *α* = 0.25 cell divisions per hour. Solid line is a fit to the distribution at 0.8 divisions per hour.

We studied the distribution of promoter activities under diverse conditions and growth rates. We find that the distributions are heavy-tailed and approximately follow a power law P(x)∼x^−2^ over two decades (Fig 2A–B). The higher the growth rate, the longer the tail of the distribution. Interestingly, we find that at a given growth rate the distributions of promoter activities are very similar under different growth conditions (Fig 2A–B and Fig S3, S4). Potential variability in translation rates and mRNA stability of GFP in the different conditions suggests that the real variability in the promoter activity distributions at a given growth rate between different conditions may in fact be even smaller than the ones observed. We find an almost identical heavy-tailed distribution when measuring the promoter activities in balanced growth (Fig S6).

The observed power-law tail is similar to that found in microarray studies that measured the distribution of gene expression [19],[20]. Note however, that the present results are for promoter activities (rate of transcript initiation), whereas microarrays measure mRNA levels which are a balance of production and degradation. In addition, the present results focus on the distribution at distinct growth rates throughout different growth conditions and phases of growth.

To begin to analyze this distribution, we focused on the distribution of promoter activities of two classes of genes: Ribosomal and metabolic genes. We find that ribosomal promoters are always at the high end of the distribution, whereas metabolism-related promoters are found at the low to mid ranges of the distribution (Fig 2). This suggests that the distributions are ‘scale rich’ [21]–[24] rather than ‘scale free’ [25],[26] in the sense that they have defined scales for the different functional classes of promoters.

Distributions of additional functional classes of genes also generally display defined scales at the low to mid ranges of the distribution (Figs S7, S8, S9, S10, S11, S12, S13, S14, S15).

**Figure 3:**
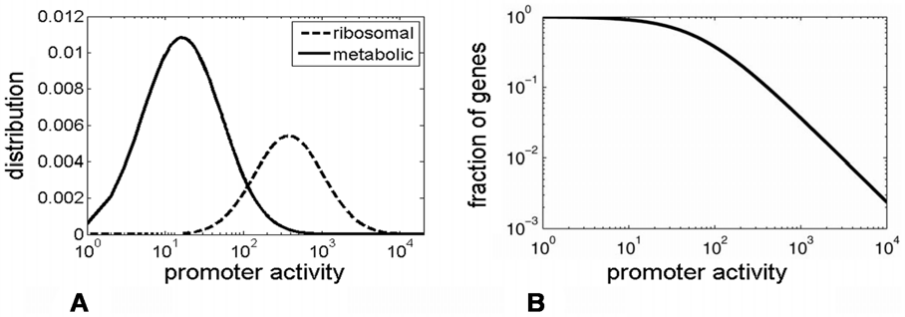
Heavy-tailed distribution obtained by a mixture of two log-normal distributions. **(A)** Log-normal distributions with the observed mean and standard deviation of ribosomal promoters (dashed) and metabolic promoters (solid line) at *α* = 0.8 divisions per hour in glucose medium. The ribosomal function was multiplied by 5 for clarity. **(B)** Rank frequency plot for the resulting mixture of these two ‘scale rich’ classes.

Interestingly, we find that a superposition of two log-normal distributions of promoters, one with low and one with high average intensities, gives rise to a combined distribution that resembles a power-law in a log-log plot over two to three decades (Fig 3). Thus the observed heavy-tail distribution might result from the sum of two (or more) distributions with defined scales.

### The relative positions of metabolic genes in the distribution change between conditions

**Figure 4:**
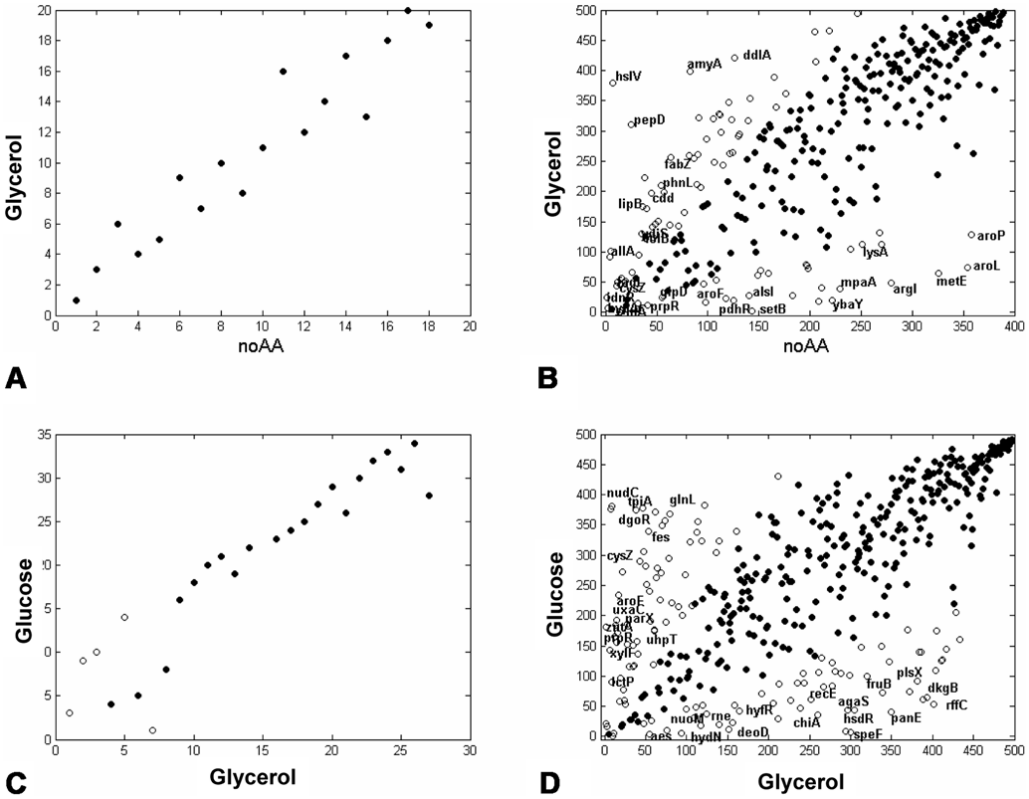
Promoter activities of ribosomal components are more correlated between conditions than metabolic promoters. Shown are the rank-rank plots of ribosomal component genes **(A,C)** and metabolic genes **(B,D)** at two pairs of conditions– Glycerol vs. no amino acids **(A,B)** and Glucose vs. Glycerol **(C,D)**. Filled circles – genes for which the ranks differed by less than twofold between the two conditions. Open circles – genes for which the rank ratio between the two conditions differed by more than twofold. Gene names for which the fold expression between conditions changed the most are displayed. All data is at a growth rate of 0.5 cell divisions per hour.

The finding that the distribution of promoter activities at a given growth rate does not depend on growth conditions may be counter-intuitive, because each condition is expected to require a different set of genes to be expressed. Indeed, we find differences in the relative compositions of expressed genes under the different growth conditions (Figs 1, 2, 4).

Not only is the total distribution invariant, but also the distributions of ribosomal and metabolic promoter activities are nearly invariant across different conditions (Fig 2). However, there is a notable difference between ribosomal promoters and promoters of metabolic genes. The activities of ribosomal promoters are rather constant from one condition to another (Fig 4A,C), whereas metabolic promoter activities vary widely across conditions (Fig 4B,D). Overall, the rank correlation across all conditions for ribosomal promoters is high (0.92+/−0.01) while metabolic promoters show significantly lower rank correlation (0.71+/−0.01) (Fig S16). In other words, the pool of metabolic genes at a given growth rate is made up of different proportions of mRNAs for each condition. For example, amino acid biosynthesis genes, such as *aroP*, *metE* and *trpL*, rank high in expression in the growth condition with no amino acids, but very low in conditions with amino acid (Fig 4B). Despite the varying composition of metabolic promoters, their summed expression seems to depend only on growth rate and not on the specific conditions (Fig S2). They are re-positioned in each condition but end up forming very similarly shaped distributions.

### Fraction of ribosomal expression grows linearly with growth rate

**Figure 5:**
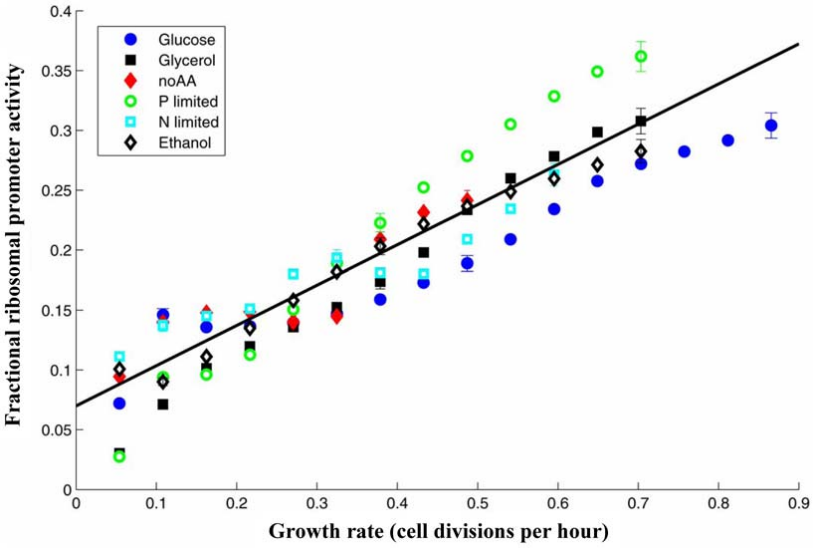
Fraction of ribosomal promoter activity increases linearly with growth rate. Shown is the sum of promoter activities of the 19 ribosomal promoters (corresponding to 63 ribosomal genes) divided by the total promoter activity of all 1,920 promoters in the library for six different conditions at 30°C. Linear regression of the data is also shown (R^2^ = 0.97±0.03). Note that at different environmental conditions the cells reach different maximal growth rates (highest in the glucose condition and lowest in the condition with no amino acids). Standard errors are shown for three representative growth rates for each condition.

Previous studies, conducted under balanced growth (deep exponential phase), demonstrated that total ribosomal fraction in bacteria cells increases linearly with growth rate [3], [4], [10]–[14]. As our system allows measuring promoter activities on a genome scale at different stages of growth ranging form exponential to stationary phase, we analyzed the fraction of total transcriptional resources allocated to ribosomal promoters. We measured the sum of the promoter activities of all 19 promoters included in the library that drive ribosomal operons (these operons contain 63 genes, making up ∼70% of known ribosomal-related promoters including ribosomal RNA and ribosomal proteins). We find that the fraction of ribosomal promoter activity out of the summed activity of all promoters increases linearly with growth rate (R^2^ = 0.97±0.03), from 7% at 0.1 cell divisions per hour to 30% at 0.7 divisions per hour (Fig 5).

Importantly, nearly the same linear curve is found for different growth conditions and phases of growth (Fig 5). For example, the ribosomal fraction of promoter activity for cells grown in the absence of amino acids depends on growth rate in the same way as cells grown with saturating levels of amino acids, despite the fact that growth in the presence of amino acids is almost twice as fast as that without amino acids (Table 1). The linear dependence applies to cells in early, mid- and late-exponential phases as well as to cells that slow growth as they enter stationary phase. Thus for a given growth rate, the fraction of promoter activity allocated to ribosomal promoters is relatively invariant to growth conditions.

The fact that the fraction of ribosomal promoter activities increases linearly with increasing growth rates can explain the more skewed distribution at higher growth rates (Fig 2). The linear dependence on growth rate was observed not only for the sum of all ribosomal components, but also when each of the components (rRNA, ribosomal proteins) was considered separately (Fig S17).

### Simple model for resource allocation suggests a linear relation between growth rate and the fraction of ribosome expression

We present a simple model that can explain the invariance of the fractional ribosomal promoter activities under a framework of optimal resource allocation. We follow the pioneering work of Ehrenberg and Kurland [9], and pose resource allocation as an optimization problem, where the cell maximizes its growth rate. We find that this optimization problem has a surprisingly simple solution that is independent of many details of the environment.

Consider a cell that has two types of proteins: ribosomal proteins *R* that make the ribosomes that produce new proteins, and metabolic proteins *P* that provide the building blocks needed for cell growth and protein synthesis. We seek the optimal partition between *R* and *P* that maximizes the cells' growth rate.

To proceed, note that cell growth under most conditions is limited by the rate of protein production. Thus one seeks to increase *R* and *P*. This cannot be done without limit, because one cannot increase the density of the cytoplasm beyond a certain value. Experiments show that there is a fixed concentration *C* of total protein [1],[6] that is invariant to conditions and growth rate. Thus, the concentrations of *R* and *P* obey the conservation law
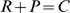
(1)The ribosomes enhance the growth rate *α* by producing proteins. For simplicity, we assume that they function as an enzyme with Hill-type kinetics that acts on a substrate *S*, for example amino acids needed for translation [9]:
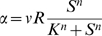
(2)In this equation, the rate of protein production is described as a Hill-function of the resource *S*. The maximal growth rate per ribosome at unlimited resources is *v*. This parameter incorporates the peptide elongation rate.

The resource *S* is provided by the metabolic proteins *P*. The proteins in *P* are typically enzymes that are in much lower concentrations than their small-molecule substrates. Hence, in this simple case, the resource that *P* provides is proportional to the concentration of *P*:
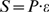
(3)where the parameter *ε* describes the availability of substrates in the environment (the growth condition). The smaller the environmental parameter* ε*, the smaller *S*, and the lower the growth rate. As we will see, this parameter will drop out of the equations and will not play a role in the optimal solution.

**Figure 6:**
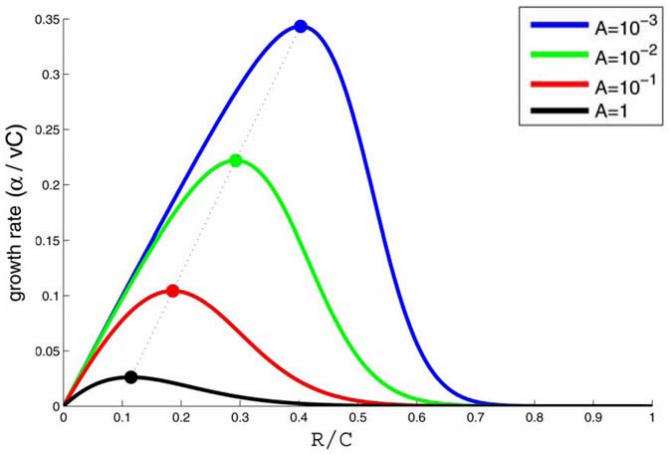
Model for resource allocation between ribosomal and metabolic proteins. Scaled growth rate (α/*vC*) as a function of the fraction of ribosomal constituents *φ = R/C*. Growth rate is maximal at intermediate levels of *φ* (filled circles). Richer environments (lower parameter *A* in the model) have higher optimal growth rates and a higher optimal *φ*. The relation between the maximal growth rate and the ribosomal fraction at which the maximum is obtained is linear (black line).

The three equations can be united to a single equation for the growth rate as a function of the fraction of ribosomal proteins, *φ = R/C*:
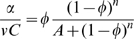
(4)where the parameter *A = (K/εC)^n^* inversely depends on the richness of the environment described by *ε*. As shown in Fig 6, in a given environment (given value of *A*), the growth rate is zero when *φ = 0*, because all proteins are non-ribosomal, *R* = 0. It is also zero at the other extreme when *φ* = 1, because the cell is full of ribosomes with no *P* proteins to provide resources for the ribosomes to work with. The growth rate has a maximum at intermediate* φ*. Different environments, represented by different values of *A*, give different optimal values *φ_opt_*.

Maximizing the growth rate with respect to *φ* provides a surprisingly simple solution. Differentiating Eq. 4 with respect to *φ* and equating to zero (the optimal solution) results in the following relation:
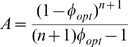
(5)Substituting (5) in (4):
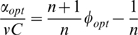
(6)Solving for the optimal fraction of ribosomes *R_opt_/C = φ_opt_* we obtain:
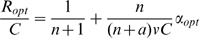
(7)


Thus the optimal fraction of ribosomes out of the total amount of proteins *(R_opt_/C)* = *φ_opt_* increases linearly with growth rate. Moreover, this relation is independent of the conditions. The same slope and intercept are found regardless of, say, the availability and nature of the sources of carbon, nitrogen, phosphate etc. in the environment. Mathematically, the optimal ribosomal fraction *R_opt_/C* in Eq 7 does not depend on the parameters *ε* or *K*. Note that the linear relation obtained by solving the model is not a result of the peptide-chain elongation rate being independent of growth rate, but rather a result of the cell being in an optimal resource allocation point.

The model can be extended to include, in addition to *R* and *P*, general constitutively expressed housekeeping proteins *E*, whose concentration does not depend on condition and growth rate. In this case, the total concentration of proteins is made up of these three groups *R+P+E = C*. The optimal resource allocation in such a model is identical to that in Eq. 7, with a linear dependence of the optimal ribosome fraction on the growth rate, except that the intercept is multiplied by *C′/C*, where *C′ = R+P*.
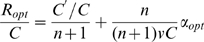
(8)To compare the model to the data, we first estimated the maximal relative fraction of both metabolic and ribosomal promoters: *C′/C* = 0.4+/−0.05 (Fig S17). Using this and the observed intercept at α = 0, *R/C* = 0.07 (Fig 5) we find that the Hill coefficient *n* which best describes the data is *n* = 6.

## Discussion

This study used a comprehensive library of reporter strains together with a robotic assay to examine the effect of growth rate on the genome-wide distribution of promoter activities in *E. coli*. We find that the distribution is heavy-tailed showing a power-law of p(x)∼x^−2^, similar to that found by DNA microarrays in yeast and fruit flies. Interestingly, we find that the distribution of promoter activities seems to be invariant of growth conditions and depends only on growth rate. This invariance is found under diverse growth conditions with different limiting nutrients and stresses, and under both exponential and post-exponential growth. A similar heavy-tailed distribution of promoter activities is found during exponential growth when cells are in balanced growth (Methods and Figs S5, S6).

The finding that the distribution of promoter activities does not change in different conditions is perhaps surprising, because one might expect different sets of genes to be turned ON and OFF in each condition. We find that indeed genes are differentially expressed in each condition, but that their expression levels still fall within the same distribution.

The distribution is scale-rich [21]–[24], containing a constant high-end of ribosomal promoters, and low-mid intensity range of metabolic promoters. The latter promoters change relative expression levels between conditions, but adhere to the same overall distribution. The two classes of promoters differ in the way their relative composition varies between different growth conditions. While the relative composition of ribosomal promoters is quite constant across different growth conditions, the relative composition of expressed metabolic promoters changes in a correlated manner to the environment. The higher variability in the relative activity of metabolic promoters may ensure that the ribosomal machinery is fed with the necessary building blocks, regardless of changes in the environment.

In the present study we use promoter activity measurements as indicators for allocation of transcriptional resources, where high transcription rates necessitate more transcriptional resources to be allocated. Since our experimental approach is based on measuring plasmid-based fluorescence, the copy number of virtually all of the promoters is equal. This, however, is not the case when considering ribosomal RNA genes which are clustered on the chromosome in seven copies. Moreover, this cluster is in proximity to the origin of replication which suggests that more than seven copies are likely to be found during exponential growth. Thus, when considering the multiple copy number of these genes, the distribution observed in Fig 2 is expected to span a wider range.

To understand the invariance in the observed scale-rich distribution we also studied the total fraction of promoter activities allocated to ribosomal promoters. We find that the fraction of ribosomal promoter activity in *E. coli* increases linearly with growth rate regardless of the composition of the growth media. The linear relation is nearly invariant to growth conditions. This can be used to explain the shape of the promoter activity distribution in terms of the sum of two (or more) gene class distribution, as shown in Fig 3. While the linear relation between ribosomal fraction and growth rate has been previously demonstrated for balanced growth [3], [4], [10]–[14], here we find a similar linear relation in non-balanced growth at the level of promoter activities.

We present a simple model that explains the invariance of the promoter activity distributions by accounting for the invariant fraction of resources allocated to the ribosomal components. The model predicts that in order to maximize growth rate, resource allocation at the optimal growth rates yields a linear relation between the fraction of ribosome components and the optimal growth rate, independently of the details of the environmental conditions. It is important to note that the model considers protein concentration units while our measurements are of promoter activity levels. This is a simplification as promoter activities should not correlate precisely with protein concentrations when considering possible post-transcriptional regulation.

Promoter activities were calculated based on measurements of growth (od) and fluorescence (GFP). In particular, the usage of a stable GFP enabled us to calculate the rate at which GFP accumulates in the cells by taking the time derivative of the fluorescence measurements. By doing so, we assumed that regulatory processes downstream to transcription (e.g. mRNA degradation, translation) are at a constant rate. While such processes may vary when conditions change throughout growth, the invariant distribution observed across all conditions suggests that such variability is minimal. Moreover, the distributions among the different conditions are always compared at a specific growth rate; thus, possible variability due to different growth conditions is probably negligible.

An interesting question is the origin of the invariant distribution of promoter activities within the class of metabolic genes. It seems that a fixed range of resources (in terms of total promoter activity) is allocated to the metabolic class of promoters. Within this fixed range of allocated resources, the relative rank of the promoters varies according to the growth condition. A model by Furusawa et al [19] suggests that this is a generic property of a class of large chemical networks. It would be interesting to seek an explanation for this invariant distribution in terms of optimal solutions of resource allocation models similar to the one presented here.

The present experimental approach, using a robotic system to assay a comprehensive library of reporter strains, opens the way for large-scale measurements of promoter activities in *E. coli* in diverse conditions and growth phases. It would be interesting to extend this study to find the underlying molecular mechanisms giving rise to the invariant distribution of promoter activities (*e.g.*, measuring the distribution in mutant backgrounds, or using drugs which prevent the cells from dividing). In particular, the experimental setup presented here may be useful in characterizing modulations in promoter activities following antibiotic treatments which were recently shown to have profound effect on the cell's metabolic state as well as on it's gene expression program [27],[28].

The platform used in this study measures the averaged promoter activity in a population of cells. An outstanding question is how the distribution of single cells within a population of a given reporter strain varies in different growth rates across different conditions [29]. Furthermore, many genes, in particular ribosomal proteins, are known to be regulated at the post transcriptional level. It would be interesting to examine if the same distribution is maintained when considering protein levels. More fundamentally, it would be interesting to explore the design constraints that lead to the observed invariant distribution shapes found in this study. The possibility that the linear relation between fractional ribosomal promoter activities and growth rate maximizes the possible growth rate suggests that strong selection forces should optimize how limited resources would be partitioned; however, the evolutionary and molecular mechanisms underlying such a global design are yet to be discovered.

## Materials and Methods

### Growth mediums

All media were based on M9 defined medium (0.6% Na_2_HPO_4_, 0.3% KH_2_PO_4_, 0.05% NaCl, 0.01% NH_4_Cl, 0.1 mM CaCl_2_, 1 mM MgSO_4_, 5·10^−4^% Thiamin). The media used in this study are: Gluocse (M9 minimal medium +0.5% glucose +0.1% Amino Acids (AA, Casein peptone, Pronadisa Ltd) +50 µg/ml kanamycin); Glycerol (M9 minimal medium +0.5% glycerol +0.1% AA +50 µg/ml kanamycin); No amino-acids (M9 minimal medium +0.5% glucose +50 µg/ml kanamycin); Phosphate limitation (M9 minimal medium diluted 1∶5 into M9 minimal medium lacking Na_2_HPO_4_ and KH_2_PO_4_ +0.5% glucose +0.1% AA +50 µg/ml kanamycin. pH was corrected to 7 using MOPS); Nitrogen limitation (M9 minimal medium diluted 1∶5 into M9 minimal medium lacking NH_4_Cl +0.5% glucose+50 µg/ml kanamycin); Ethanol (Glucose medium +4% absolute ethanol +50 µg/ml kanamycin). We chose the 4% ethanol condition since preliminary assays showed that *E. coli* cells can grow in up to 6% ethanol without compromising viability (although growth rate is considerably reduced, Fig S18). Note that growth rates of individual promoters exhibit a plateau during exponential growth (Fig S19).

### Robotic assay for genome-wide promoter activity

The library of reporter strains, each bearing a low-copy plasmid with a promoter of interest controlling fast-folding GFP (GFPmut2 [30]) was previously described [16]. Reporter strains were inoculated from frozen stocks and grown over-night on glucose medium for 16 hours in high-brim 96-well plates. The 96-well plates were covered with breathable sealing films (Excel Scientific Inc.). All steps from this point were carried out using a programmable robotic system (Freedom Evo, Tecan Inc.). Overnight cultures were first diluted 1∶10 into the glucose medium followed by a second 1∶10 dilution into one of the growth media. The second dilution was done into black non-coated 384-well plates with optical flat bottom (Nunc), which were used for continuous cells growth. The final volume of the cultures in each well was 60 µl. A 20 µl layer of mineral oil (Sigma) was added on top to avoid evaporation. The plates were inserted into a temperature-controlled shaker station. A robotic arm moved the 384-well plates from the incubator-shaker to the plate reader (Infinite F200, Tecan Inc.) and back. Optical density (600 nm) and fluorescence (535 nm) were thus measured periodically at intervals of 16 minutes over 14 h of growth. The temperature in the incubator-shaker and in the reader was set to 30°C.

We note that anaerobic conditions may arise when growing cells in small tubes (384-well plates). However, the fact that a power law distribution, in which ribosomal genes make up the higher end, is observed during well-aerated balanced growth as well (Fig S6), suggests that this is probably a general design principle rather then an experimental artifact. In addition, anaerobic conditions which may affect GFP fluorescence are likely to develop in all cell cultures in a given condition. Any such effect will equally affect the different reporter strains and therefore will cancel out.

Although changes in growth rate affect the plasmid copy number in the reporter strains [31], these modulations do not affect our analysis since all library strains are based on the same backbone-vector with the same origin of replication. Thus, modulations of growth rates which lead to plasmid copy number changes are likely to occur equally in all reporter strains. These changes will eventually scale proportionally with the measured expression levels in all reporter strains.

To ensure that reporter strains with high GFP expression do not show slower growth rate we analyzed the correlation between growth rate and GFP expression levels for individual strains. We find no correlation between maximal growth rate and maximal promoter activity of the strains (correlation coefficient = −0.007, p = 0.75). Furthermore, *rpsL*, a ribosomal reporter strain (one of strongest promoters in the library), and a promoterless strain (which makes no GFP) grow in almost identical rate during balanced growth as can be seen in Fig S5.

### Data analysis

Data was automatically obtained from the robot software (Evoware, Tecan) and processed using custom Matlab software. All OD and GFP measurements were background subtracted separately for each overnight 96-well plate cultures. Outlier cultures in which OD curves deviated more than three standard deviation of the mean OD curve for the plate, were discarded (less than 5% of cultures). For each 96-well plate, a background GFP curve was constructed by the mean of the 15% of the cultures with lowest GFP readings. These bottom 15% usually included the two strains with promoterless vector used as controls in each 96-well plate. Strains whose GFP curve was below 2 standard deviations above this background curve were considered to have undetectable promoter activity. Promoter activity was calculated as the temporal derivative of the background subtracted GFP intensity divided by the OD, PA = dGFP/dt/OD [16]. Growth rate was calculated as the temporal derivative of the natural logarithm of the OD curves, α = *dln(OD)/dt*. We considered only growth rates which were reached by at least 90% of the cultures in a given condition. Identities of ribosomal and metabolic proteins were according to the physiological role annotations of Ecocyc version 8.5 [32]. Fig S20 presents the same data as shown in Fig 1 but the order of the genes is sorted by the maximal level of the promoter activities. All the data can be found in the Supporting Information datasets S1, S2, S3. Promoter activities measured in this work are averages over a population of cells. FACS measurements performed on each strain generally show a uni-modal distribution, with no apparent sub-population structure (data not shown).

#### Error analysis

Error bars were estimated as follows – given the standard error of 20%, estimated from the repeated strains(Fig S1), we estimated the standard error of average ribosomal promoter activity as 
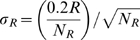
 where N_R_ is the number of ribosomal promoters, and the standard error of the average total promoter activity as 
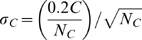
 where N_C_ is the total number promoters. The standard error in the estimation of R/C follows from the law of propagation of errors: 
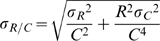
.

### Measuring promoter activity during balanced growth

We chose a subset of reporter strains with different promoter activities that together span the entire range of the power law distribution as observed during non-balanced growth in 384-well plates. This subset included 4 ribosomal genes and 28 metabolic genes. We measured promoter activity in these strains under two conditions: (1) glucose condition and (2) no amino acids condition, as described for the assays done with 384-well plates. To achieve well-aerated balanced growth, over night cultures were diluted 1∶400 and grown in wide-mouth glass tubes (15 mm width) with vigorous shake (250 rpm, 30°C). Growth was monitored by OD (600 nm) and both OD and GFP (485/535 nm) measurements were taken during exponential growth. OD and GFP were measured by removing 150 µl from the batch culture and placing in 96-well plates (Nunc) which were then assayed using Victor3 plate reader (Perkin Elmer). Promoter activity was measured by taking the time derivative of the GFP divided by OD PA = dGFP/dt/OD [16].

## Supporting Information

Figure S1 Reproducibility of promoter activity measurements. Shown are the Promoter activities of 21 identical repeats of two control strains - *wrbA* and *serA*, each run on a different plate (average is shown in black). The bottom plots show all pairwise comparisons between these sets.
(0.19 MB TIF)

Figure S2 Total promoter activity is relatively constant between growth conditions but strongly dependent on growth rate. Shown is the average over all growth conditions of the sum of the promoter activities at different growth rates. (a) All promoters. (b) Metabolism related promoters. Standard errors are over the different growth conditions.
(0.04 MB TIF)

Figure S3 Rank-frequency plots of promoter activities for the six growth conditions of Fig 1. Horizontal axis is the promoter activity levels at a given growth rate; Vertical axis is the fraction of promoters with equal or higher promoter activity level. Black points - all genes; Empty green - ribosomal promoters; Solid red - metabolic proteins. X - glucose medium, Circles - ethanol, diamonds - glycerol, squares - no amino-acids, V -Phosphate limitation, triangles - Nitrogen limitation. (a) Data at α = 0.8 cell divisions per hour. (b) Data at α = 0.25 cell divisions per hour. Dashed line is a fit to the distribution at 0.8 cell divisions per hour.
(0.11 MB TIF)

Figure S4 Rank-frequency plots of promoter activities for the six growth conditions of Fig 1. Horizontal axis is the promoter activity levels at a given growth rate; Vertical axis is the fraction of promoters with equal or higher promoter activity level. Blue - glucose medium, green - ethanol, red - glycerol, cyan - no amino-acids, magenta -Phosphate limitation, black - Nitrogen limitation. (a) Data at α = 0.8 cell divisions per hour. (b) Data at α = 0.25 cell divisions per hour. Dashed line is a fit to the distribution at 0.8 cell divisions per hour.
(0.11 MB TIF)

Figure S5 Growth rate of two representative reporter strains during balanced growth (a) in GLU condition (b) in no amino acids condition. Blue, promoterless strain; Red, *rpsL*.
(0.10 MB TIF)

Figure S6 Rank-frequency plots of promoter activities for 32 reporter strains in two conditions (a) GLU conditions (b) in no amino acids condition. The strains were grown in well-aerated glass tubes so that balanced growth was reached. The distributions were fitted to a power law distribution and the best fit results in the following exponents: (a) GLU condition; α = −1.87. (b) No amino acids condition; α = −2.2. These values are very similar to the values that best fit the distribution observed during non-balanced growth using 384-well plates (α∼−2).
(0.03 MB TIF)

Figure S7 Rank frequency plot of motility, chemotaxis, energytaxis genes. Blue - glucose medium, green - ethanol, red - glycerol, cyan - no amino-acids, magenta - Phosphate limitation, black - Nitrogen limitation.
(0.11 MB TIF)

Figure S8 Rank frequency plot of SOS response genes. Blue - glucose medium, green - ethanol, red - glycerol, cyan - no amino-acids, magenta -Phosphate limitation, black - Nitrogen limitation.
(0.10 MB TIF)

Figure S9 Rank frequency plot of TCA cycle genes. Blue - glucose medium, green - ethanol, red - glycerol, cyan - no amino-acids, magenta -Phosphate limitation, black - Nitrogen limitation.
(0.10 MB TIF)

Figure S10 Rank frequency plot of drug response/sensitivity genes. Blue - glucose medium, green - ethanol, red - glycerol, cyan - no amino-acids, magenta -Phosphate limitation, black - Nitrogen limitation.
(0.10 MB TIF)

Figure S11 Rank frequency plot of cell division genes. Blue - glucose medium, green - ethanol, red - glycerol, cyan - no amino-acids, magenta -Phosphate limitation, black - Nitrogen limitation.
(0.10 MB TIF)

Figure S12 Rank frequency plot of house keeping genes. These are genes that had an expression level above background in all six conditions studied (ribosomal components were excluded). Blue - glucose medium, green - ethanol, red - glycerol, cyan - no amino-acids, magenta -Phosphate limitation, black - Nitrogen limitation.
(0.10 MB TIF)

Figure S13 Rank frequency plot of anaerobic respiration genes. Blue - glucose medium, green - ethanol, red - glycerol, cyan - no amino-acids, magenta -Phosphate limitation, black - Nitrogen limitation.
(0.10 MB TIF)

Figure S14 Rank frequency plot of aerobic respiration genes. Blue - glucose medium, green - ethanol, red - glycerol, cyan - no amino-acids, magenta -Phosphate limitation, black - Nitrogen limitation.
(0.10 MB TIF)

Figure S15 Rank frequency plot of transport genes. Blue - glucose medium, green - ethanol, red - glycerol, cyan - no amino-acids, magenta -Phosphate limitation, black - Nitrogen limitation.
(0.10 MB TIF)

Figure S16 Promoter activities of ribosomal components are more correlated between conditions than metabolic promoters. Average over all pairs of conditions between the correlation coefficient of ranks for metabolic promoters (814 promoters) and ribosomal and tRNA promoters (19 promoters constituting 63 genes, making up ∼70% of known ribosomal-related promoters including ribosomal RNA and ribosomal proteins).
(0.06 MB TIF)

Figure S17 Fractional promoter activity vs. growth rate of (a) the sum C′ of ribosomal promoters R and promoters of metabolic proteins P (as defined in Ecocyc [4]). Metabolic promoters which were expressed under all conditions were excluded, since they may be considered as constitutive housekeeping proteins (included in the protein class denoted E in the model). (b) ribosomal protein promoters (c) ribosomal RNA promoters and (d) tRNA promoters. Experiments were at 30C. Blue filled circles - glucose medium, black filled squares - glycerol, red filled diamonds - no amino acids, green empty circles - phosphate limited, empty cyan squares - nitrogen limited, empty black diamonds - 4% ethanol.
(0.13 MB TIF)

Figure S18 Growth curves of *E. coli* cells in the presence of different concentrations of ethanol. The cells were grown overnight in M9 minimal medium +0.5% glucose +0.1% amino acids and diluted 1∶100 on the day of the assay into the same medium into which ethanol was added (1%, 3% and 6%). The assay was performed using flat-bottom black optical 384-well plates. Note that in this study we chose to use 4% ethanol in the growth medium.
(0.04 MB TIF)

Figure S19 Examples of OD measurements and calculated growth rates for six representative genes, demonstrating a plateau during exponential phase.
(1.26 MB TIF)

Figure S20 Normalized promoter activities sorted according to maximal level. Each row holds the promoter activities of one promoter (normalized between 0 and 1) as in Figure 1, sorted from low (top) to high (bottom) activities.
(0.84 MB TIF)

Dataset S1 All promoter activities and OD at each condition.
(21.49 MB XLS)

Dataset S2 All of the data at α = 0.8 and α = 0.25 divisions per hour, including mean, standard deviation and CV for all values greater than zero.
(0.39 MB XLS)

Dataset S3 Annotation classes of each gene.
(2.98 MB XLS)
