# Supplementary figures and images for: Invariant Distribution of Promoter Activities in Escherichia coli
Source: PLoS Comput Biol. 2009 Oct 23;5(10):e1000545. doi: 10.1371/journal.pcbi.1000545 (PMC2758578; doi:10.1371/journal.pcbi.1000545)

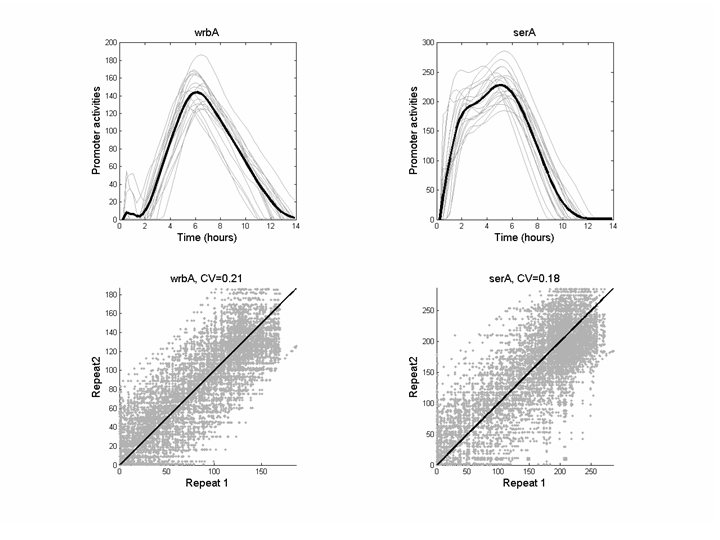

Supplement: Figure S1 — Reproducibility of promoter activity measurements. Shown are the Promoter activities of 21 identical repeats of two control strains - wrbA and serA, each run on a different plate (average is shown in black). The bottom plots show all pairwise comparisons between these sets. (0.19 MB TIF) [file pcbi.1000545.s001.tif]

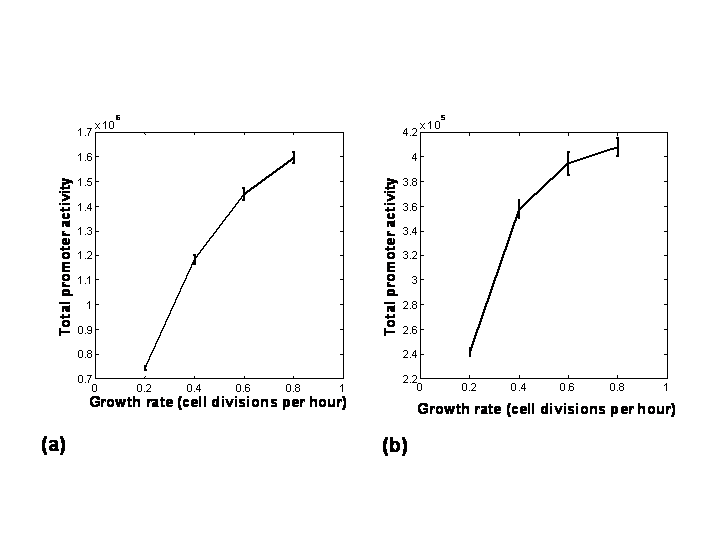

Supplement: Figure S2 — Total promoter activity is relatively constant between growth conditions but strongly dependent on growth rate. Shown is the average over all growth conditions of the sum of the promoter activities at different growth rates. (a) All promoters. (b) Metabolism related promoters. Standard errors are over the different growth conditions. (0.04 MB TIF) [file pcbi.1000545.s002.tif]

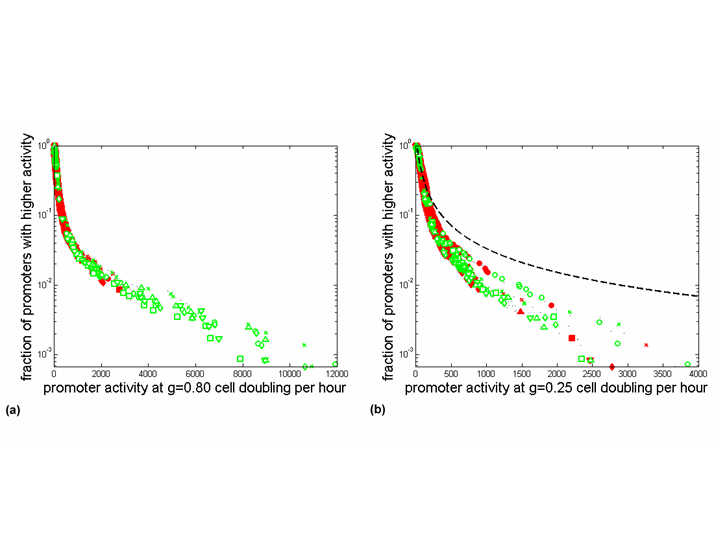

Supplement: Figure S3 — Rank-frequency plots of promoter activities for the six growth conditions of Fig 1. Horizontal axis is the promoter activity levels at a given growth rate; Vertical axis is the fraction of promoters with equal or higher promoter activity level. Black points - all genes; Empty green - ribosomal promoters; Solid red - metabolic proteins. X - glucose medium, Circles - ethanol, diamonds - glycerol, squares - no amino-acids, V -Phosphate limitation, triangles - Nitrogen limitation. (a) Data at α = 0.8 cell divisions per hour. (b) Data at α = 0.25 cell divisions per hour. Dashed line is a fit to the distribution at 0.8 cell divisions per hour. (0.11 MB TIF) [file pcbi.1000545.s003.tif]

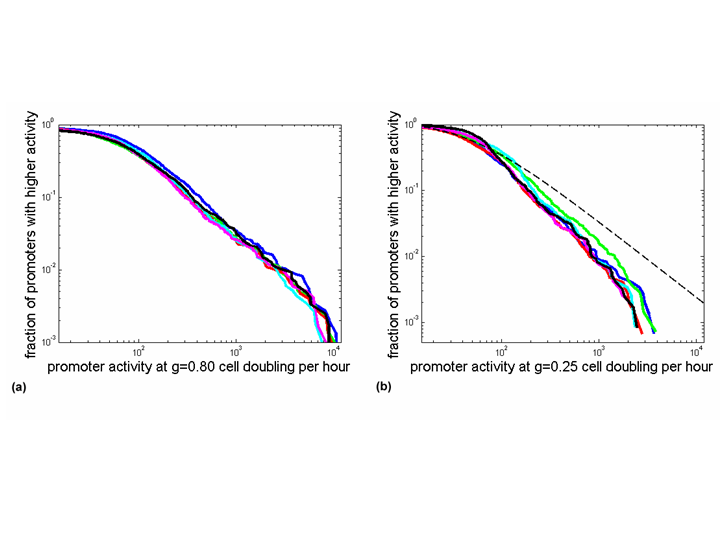

Supplement: Figure S4 — Rank-frequency plots of promoter activities for the six growth conditions of Fig 1. Horizontal axis is the promoter activity levels at a given growth rate; Vertical axis is the fraction of promoters with equal or higher promoter activity level. Blue - glucose medium, green - ethanol, red - glycerol, cyan - no amino-acids, magenta -Phosphate limitation, black - Nitrogen limitation. (a) Data at α = 0.8 cell divisions per hour. (b) Data at α = 0.25 cell divisions per hour. Dashed line is a fit to the distribution at 0.8 cell divisions per hour. (0.11 MB TIF) [file pcbi.1000545.s004.tif]

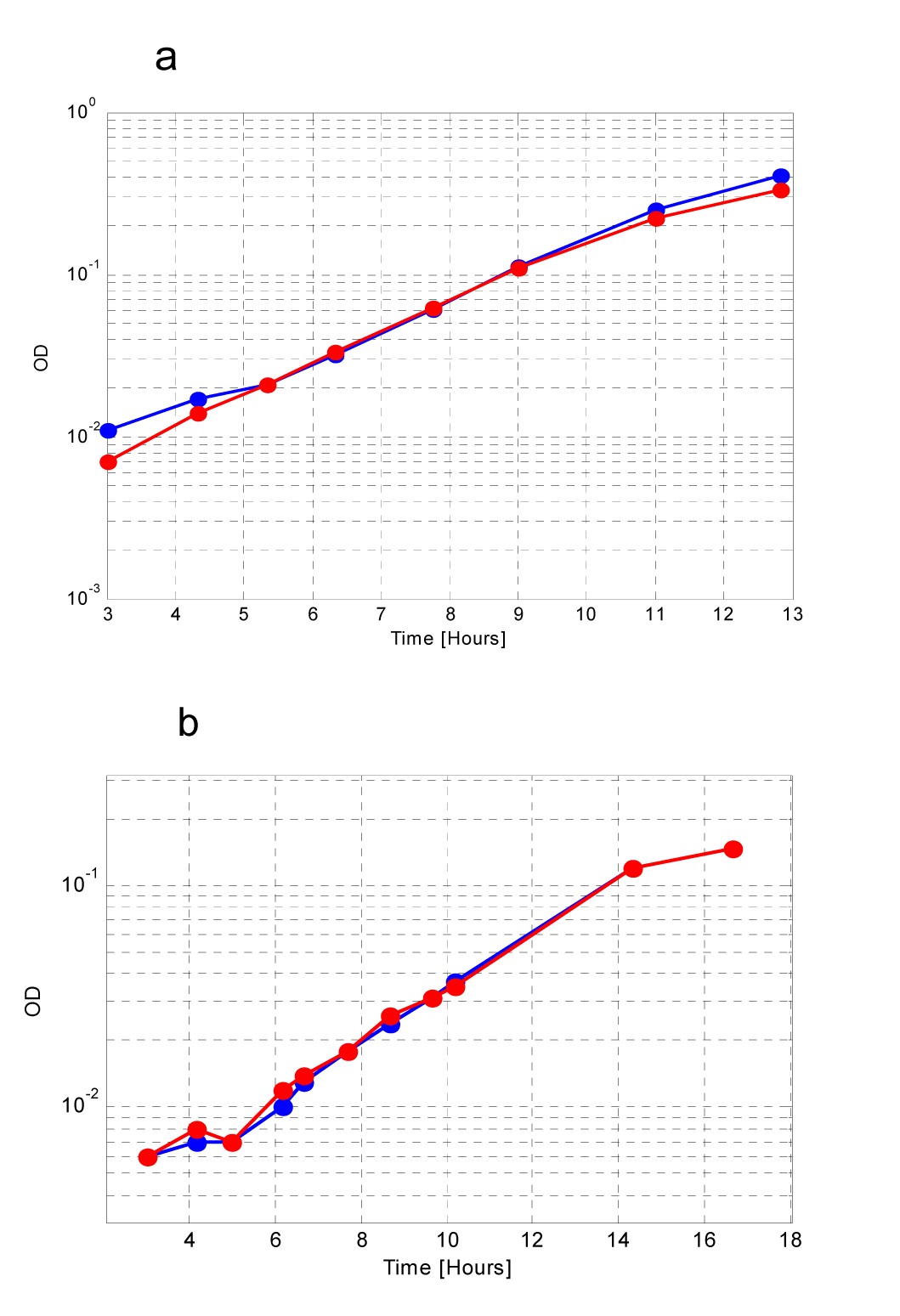

Supplement: Figure S5 — Growth rate of two representative reporter strains during balanced growth (a) in GLU condition (b) in no amino acids condition. Blue, promoterless strain; Red, rpsL. (0.10 MB TIF) [file pcbi.1000545.s005.tif]

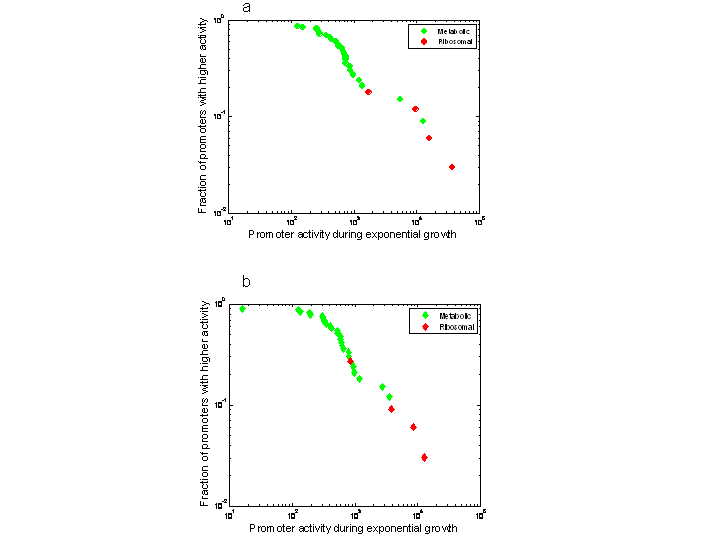

Supplement: Figure S6 — Rank-frequency plots of promoter activities for 32 reporter strains in two conditions (a) GLU conditions (b) in no amino acids condition. The strains were grown in well-aerated glass tubes so that balanced growth was reached. The distributions were fitted to a power law distribution and the best fit results in the following exponents: (a) GLU condition; α = −1.87. (b) No amino acids condition; α = −2.2. These values are very similar to the values that best fit the distribution observed during non-balanced growth using 384-well plates (α∼−2). (0.03 MB TIF) [file pcbi.1000545.s006.tif]

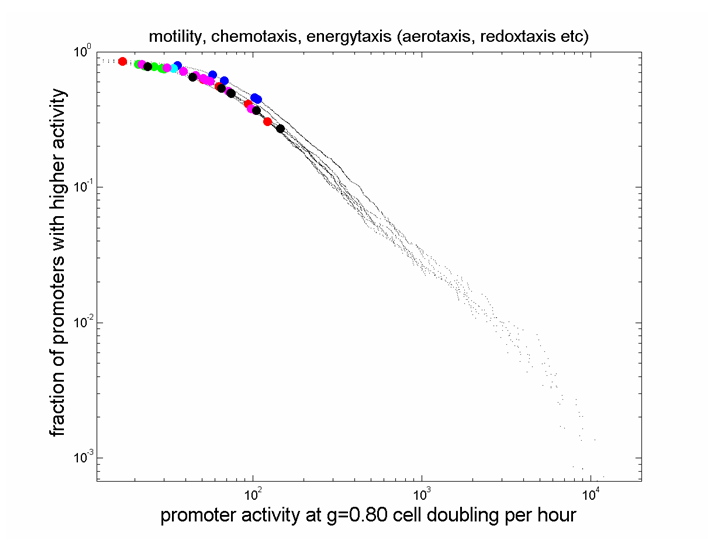

Supplement: Figure S7 — Rank frequency plot of motility, chemotaxis, energytaxis genes. Blue - glucose medium, green - ethanol, red - glycerol, cyan - no amino-acids, magenta - Phosphate limitation, black - Nitrogen limitation. (0.11 MB TIF) [file pcbi.1000545.s007.tif]

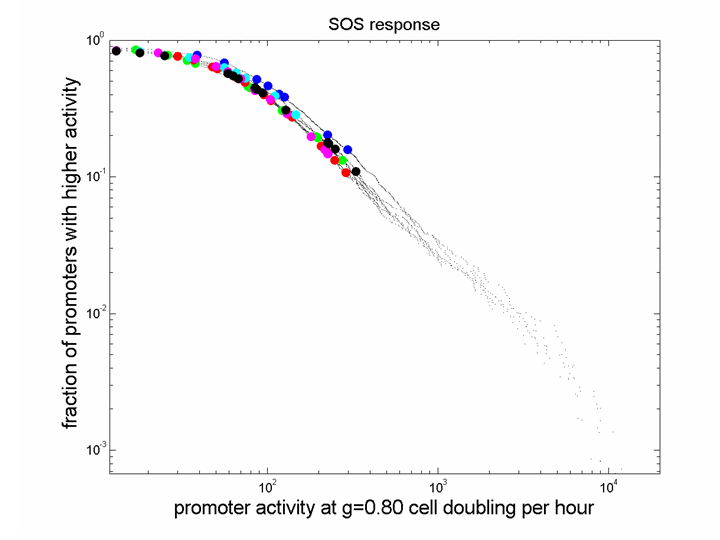

Supplement: Figure S8 — Rank frequency plot of SOS response genes. Blue - glucose medium, green - ethanol, red - glycerol, cyan - no amino-acids, magenta -Phosphate limitation, black - Nitrogen limitation. (0.10 MB TIF) [file pcbi.1000545.s008.tif]

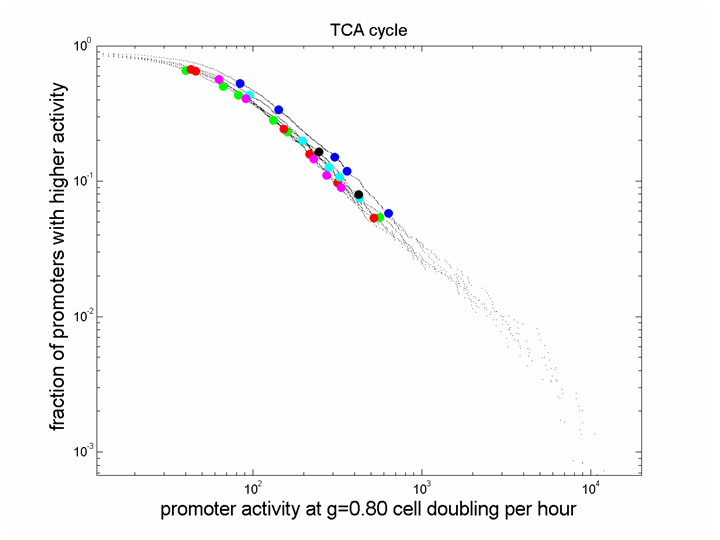

Supplement: Figure S9 — Rank frequency plot of TCA cycle genes. Blue - glucose medium, green - ethanol, red - glycerol, cyan - no amino-acids, magenta -Phosphate limitation, black - Nitrogen limitation. (0.10 MB TIF) [file pcbi.1000545.s009.tif]

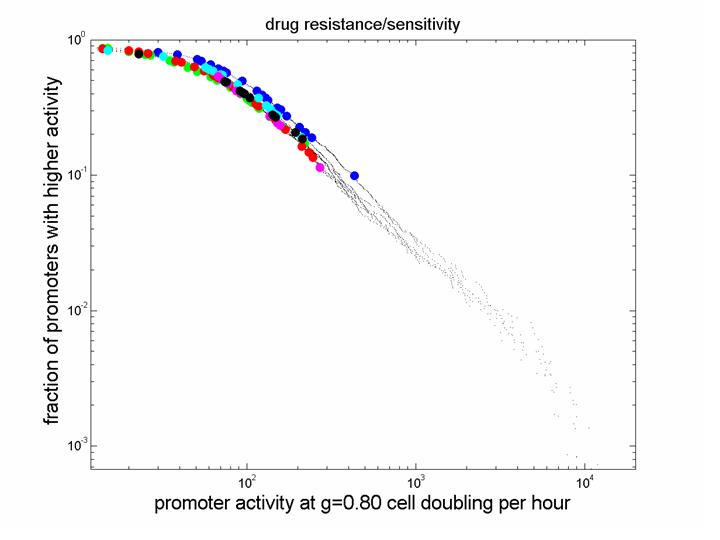

Supplement: Figure S10 — Rank frequency plot of drug response/sensitivity genes. Blue - glucose medium, green - ethanol, red - glycerol, cyan - no amino-acids, magenta -Phosphate limitation, black - Nitrogen limitation. (0.10 MB TIF) [file pcbi.1000545.s010.tif]

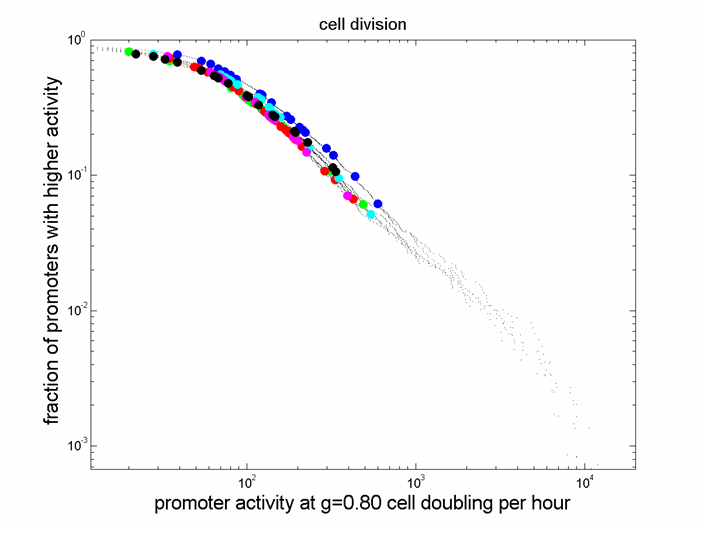

Supplement: Figure S11 — Rank frequency plot of cell division genes. Blue - glucose medium, green - ethanol, red - glycerol, cyan - no amino-acids, magenta -Phosphate limitation, black - Nitrogen limitation. (0.10 MB TIF) [file pcbi.1000545.s011.tif]

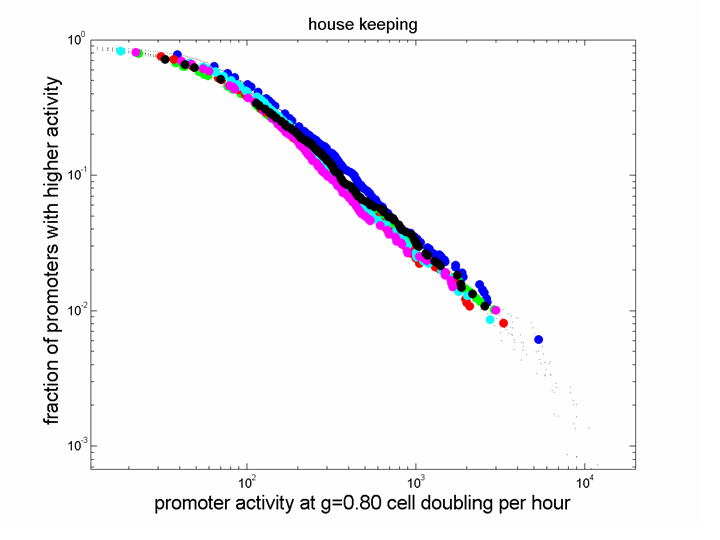

Supplement: Figure S12 — Rank frequency plot of house keeping genes. These are genes that had an expression level above background in all six conditions studied (ribosomal components were excluded). Blue - glucose medium, green - ethanol, red - glycerol, cyan - no amino-acids, magenta -Phosphate limitation, black - Nitrogen limitation. (0.10 MB TIF) [file pcbi.1000545.s012.tif]

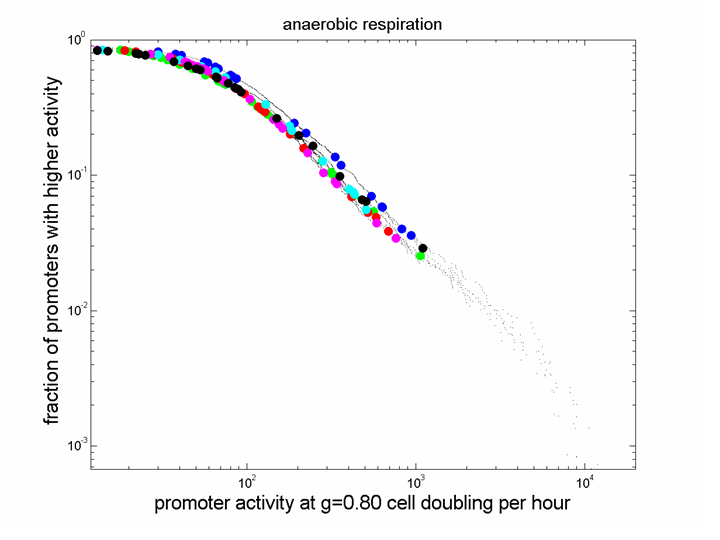

Supplement: Figure S13 — Rank frequency plot of anaerobic respiration genes. Blue - glucose medium, green - ethanol, red - glycerol, cyan - no amino-acids, magenta -Phosphate limitation, black - Nitrogen limitation. (0.10 MB TIF) [file pcbi.1000545.s013.tif]

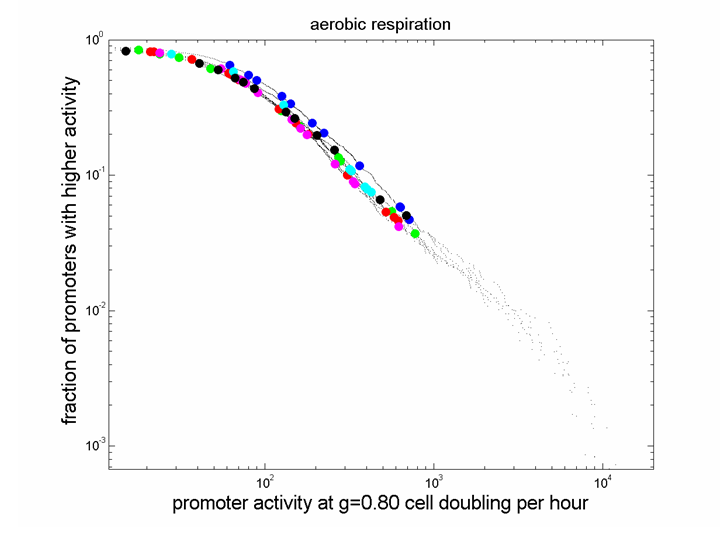

Supplement: Figure S14 — Rank frequency plot of aerobic respiration genes. Blue - glucose medium, green - ethanol, red - glycerol, cyan - no amino-acids, magenta -Phosphate limitation, black - Nitrogen limitation. (0.10 MB TIF) [file pcbi.1000545.s014.tif]

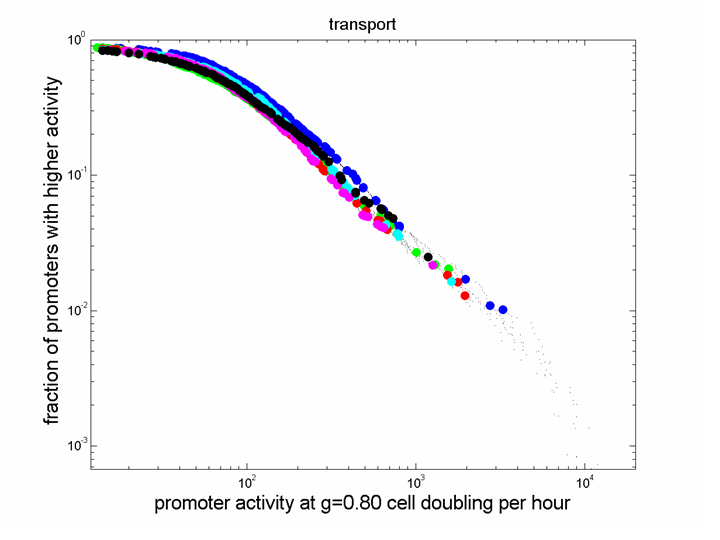

Supplement: Figure S15 — Rank frequency plot of transport genes. Blue - glucose medium, green - ethanol, red - glycerol, cyan - no amino-acids, magenta -Phosphate limitation, black - Nitrogen limitation. (0.10 MB TIF) [file pcbi.1000545.s015.tif]

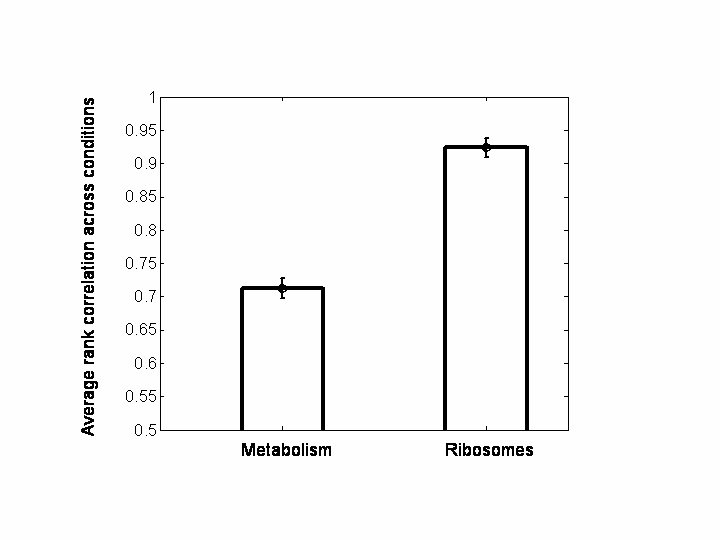

Supplement: Figure S16 — Promoter activities of ribosomal components are more correlated between conditions than metabolic promoters. Average over all pairs of conditions between the correlation coefficient of ranks for metabolic promoters (814 promoters) and ribosomal and tRNA promoters (19 promoters constituting 63 genes, making up ∼70% of known ribosomal-related promoters including ribosomal RNA and ribosomal proteins). (0.06 MB TIF) [file pcbi.1000545.s016.tif]

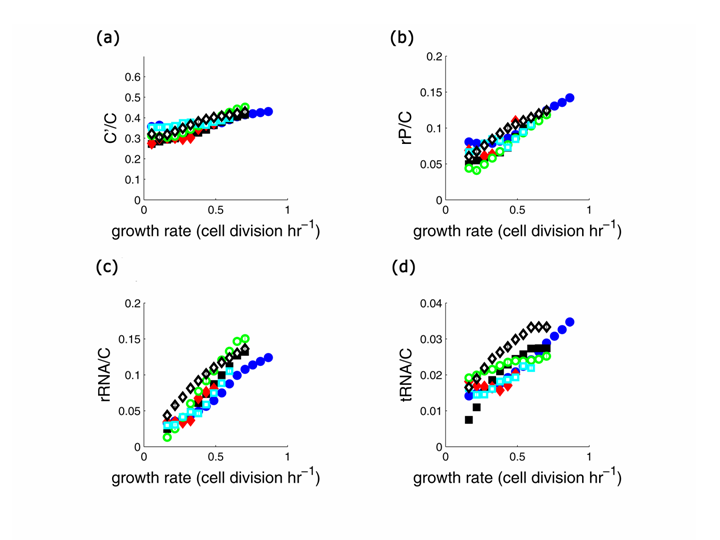

Supplement: Figure S17 — Fractional promoter activity vs. growth rate of (a) the sum C′ of ribosomal promoters R and promoters of metabolic proteins P (as defined in Ecocyc [4]). Metabolic promoters which were expressed under all conditions were excluded, since they may be considered as constitutive housekeeping proteins (included in the protein class denoted E in the model). (b) ribosomal protein promoters (c) ribosomal RNA promoters and (d) tRNA promoters. Experiments were at 30C. Blue filled circles - glucose medium, black filled squares - glycerol, red filled diamonds - no amino acids, green empty circles - phosphate limited, empty cyan squares - nitrogen limited, empty black diamonds - 4% ethanol. (0.13 MB TIF) [file pcbi.1000545.s017.tif]

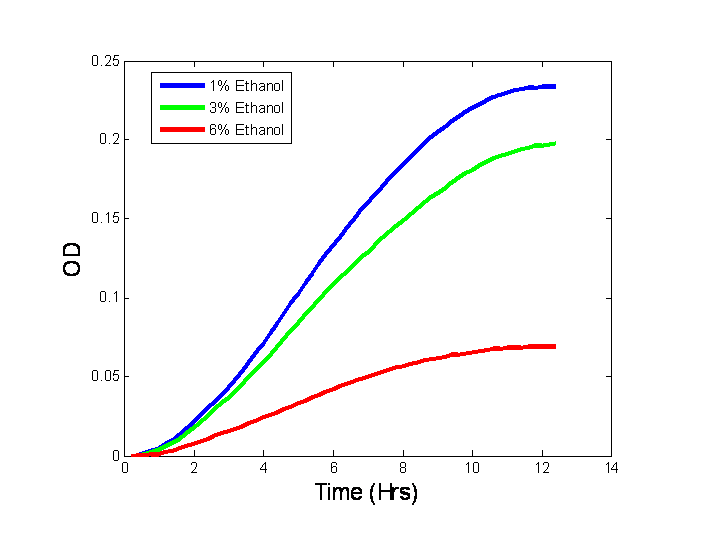

Supplement: Figure S18 — Growth curves of E. coli cells in the presence of different concentrations of ethanol. The cells were grown overnight in M9 minimal medium +0.5% glucose +0.1% amino acids and diluted 1∶100 on the day of the assay into the same medium into which ethanol was added (1%, 3% and 6%). The assay was performed using flat-bottom black optical 384-well plates. Note that in this study we chose to use 4% ethanol in the growth medium. (0.04 MB TIF) [file pcbi.1000545.s018.tif]

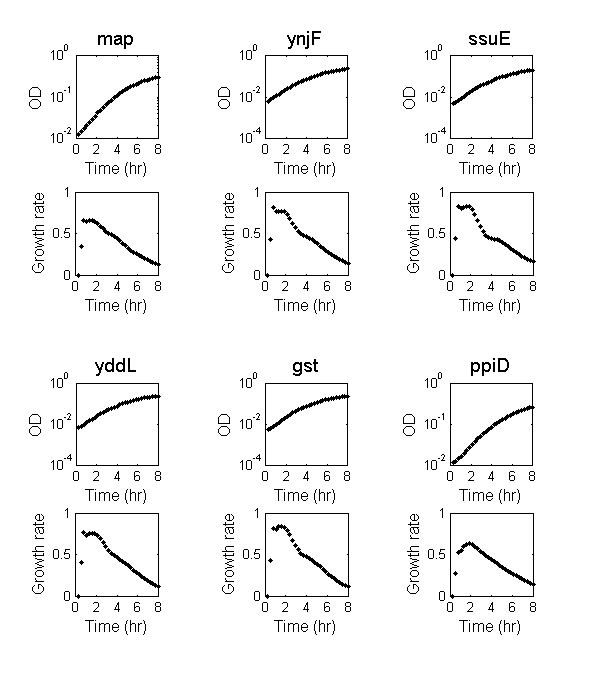

Supplement: Figure S19 — Examples of OD measurements and calculated growth rates for six representative genes, demonstrating a plateau during exponential phase. (1.26 MB TIF) [file pcbi.1000545.s019.tif]

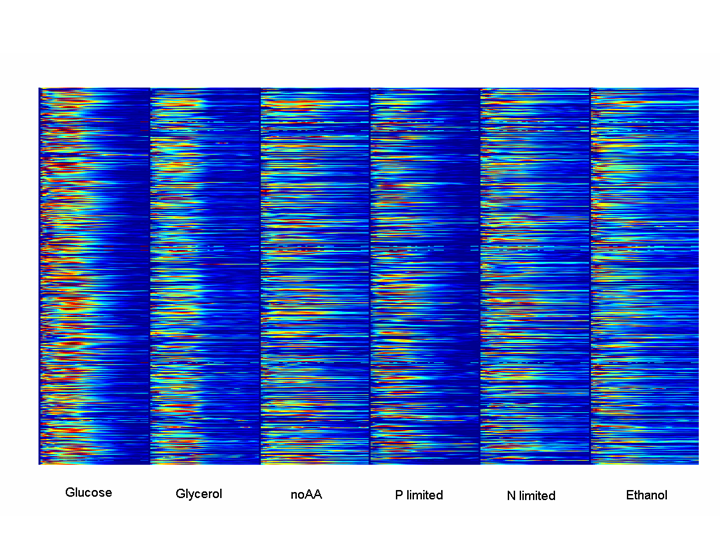

Supplement: Figure S20 — Normalized promoter activities sorted according to maximal level. Each row holds the promoter activities of one promoter (normalized between 0 and 1) as in Figure 1, sorted from low (top) to high (bottom) activities. (0.84 MB TIF) [file pcbi.1000545.s020.tif]
